# The association between polypharmacy and medication regimen complexity and antibiotic use in bronchiectasis

**DOI:** 10.1007/s11096-018-0681-1

**Published:** 2018-07-09

**Authors:** Maureen Spargo, Cristín Ryan, Damian Downey, Carmel Hughes

**Affiliations:** 1 School of Pharmacy, Queen’s University Belfast, 97 Lisburn Rd, Belfast, BT9 7BL UK; 2 The School of Pharmacy and Pharmaceutical Sciences, Panoz Institute, Trinity College Dublin, Dublin 2, Ireland; 3 Centre for Experimental Medicine, Queen’s University Belfast, Belfast, UK; 4 Regional Respiratory Centre, Belfast City Hospital, Belfast, UK

**Keywords:** Antibiotics, Bronchiectasis, Ireland, Medication regimen complexity, Polypharmacy, Pulmonary exacerbations, Treatment burden, United Kingdom

## Abstract

*Background* Polypharmacy is associated with an increased risk of adverse drug events, inappropriate prescribing and medication errors. People with bronchiectasis have frequent pulmonary exacerbations that require antibiotic therapy. *Objective* This study aimed to measure polypharmacy and medication regimen complexity in bronchiectasis patients and to explore associations between these factors and oral and intravenous (IV) antibiotic use for suspected pulmonary exacerbations. *Setting* Patients were sampled from the Regional Bronchiectasis Clinic at the Belfast Health and Social Care Trust, Northern Ireland. *Method* Data on medicines were collected from patients’ records and used to measure polypharmacy using three thresholds (≥ 4, ≥ 10, and ≥ 15 medicines’). Medication regimen complexity was calculated using the medication regimen complexity index (MRCI). Data analysis investigated differences in outcomes across polypharmacy thresholds and correlations with MRCI. *Main outcome measure* Primary outcomes were prescriptions for oral antibiotics and IV antibiotics, in the past 6 months and 2 years, respectively. *Results* Over three-quarters of the sample (N = 95) were prescribed ≥ 4 medicines (n = 74; 77.9%), 31 patients were prescribed ≥ 10 medicines (33.0%), and 12 patients (12.8%) were prescribed ≥ 15 medicines. The median MRCI was 26. Patients prescribed ≥ 10 medicines were over three times more likely to have had an IV antibiotic in the past 2 years (adjusted odd ratio 3.44, 95% confidence intervals 1.15–10.31). *Conclusion* There were significant differences in all outcomes across the ‘≥ 10 medicines’ threshold. MRCI was positively correlated with oral and IV antibiotic usage. These findings also suggest a possible link between polypharmacy and medicines regimen complexity, and poorer outcomes.

**Electronic supplementary material:**

The online version of this article (10.1007/s11096-018-0681-1) contains supplementary material, which is available to authorized users.

## Impacts on practice

- Health professionals caring for people with bronchiectasis should be vigilant to the potential impact of polypharmacy and complex medication regimens on clinical outcomes, such as pulmonary exacerbations, and take appropriate action to optimise treatment where possible.
- Regular medication review by a pharmacist in people with bronchiectasis who are experiencing frequent pulmonary exacerbations may offer a low-risk solution to a potentially high-risk problem, and could be incorporated into routine practice without delay.
- The prescription of 10 or more medicines may be an appropriate threshold to prompt medication review in patients with bronchiectasis.

## Introduction

Polypharmacy is the concurrent use of multiple prescribed medicines in one individual [1]. It is associated with an increased risk of adverse drug events, inappropriate prescribing and medication errors [1, 2]. Polypharmacy contributes to increased treatment burden—defined as a ‘dynamic multidimensional concept that is comprised of both subjective and objective elements’—of which poor adherence and suboptimal health outcomes are likely consequences [3]. There is no consensus as to how many medicines constitute polypharmacy; most sources cite a minimum of four medicines [4]. The prescription of more than 10 medicines has been described using the term ‘excessive polypharmacy’ [5]. Guidance from the National Institute for Health and Care Excellence (NICE) uses polypharmacy as a marker for identifying people with multimorbidity [6]. The guidance provides two distinct thresholds (10 or more, and 15 or more medicines) for when multimorbidity is likely to become problematic and in need of intervention [7].

The complexity of treatment is also believed to contribute to the treatment burden imposed on patients [3]. Medication regimen complexity (MRC) is the presence and combination of different dosage forms and frequencies in a person’s medication regimen. The most frequently used validated tool for measuring complexity is the Medication Regimen Complexity Index (MRCI) [4], which uses three ‘facets of complexity’ to quantify complexity [8]. The MRCI has been proposed as a risk assessment tool for identifying patients who would be suitable for medication review and intervention [9, 10].

People with bronchiectasis have frequent pulmonary exacerbations, which are treated acutely with short courses (14 days) of antibiotics [11]. Oral preparations are typically used first line, with intravenous (IV) antibiotics reserved for patients who fail to respond to oral treatment, exacerbations caused by resistant microorganisms and severe exacerbations [11].

To the best of the authors’ knowledge, the prescribing of polypharmacy and complexity of medication regimens in patients with bronchiectasis has not been investigated before.

## Aim of the study

This study aimed to determine the extent of polypharmacy and MRC in patients with bronchiectasis and to explore associations between these two factors and the prescription of oral and IV antibiotics for the treatment of suspected pulmonary exacerbations.

## Ethics approval

This study was reviewed by the Quality and Audit Department at the Belfast Health and Social Care Trust (BHSCT) on 27th July 2016, judged to be an audit and approved as such.

## Method

### Study design

This study was a retrospective observational study of the number and complexity of medicines prescribed, and antibiotic use (oral and IV), in a sample of patients with bronchiectasis. Consecutive sampling was used to select a target sample size of approximately 100 patients. Patients aged 18 years or older and who had been radiologically diagnosed with non-cystic fibrosis bronchiectasis. Patients were sampled in the order they had attended the Regional Bronchiectasis Clinic at BHSCT, Northern Ireland.

### Data collection

All data were collected by a researcher (MS) from patients’ electronic care records (ECRs) between 28th July 2016 and 4th October 2016, as close to the patients’ clinic visits as possible. Demographic and disease related data included age, gender, number of comorbidities, forced expiratory volume in 1 s (FEV_1_; percentage predicted) and latest sputum microbiology. The Charlson Comorbidity Index (CCI), a clinical risk assessment tool, was used to quantify the burden of certain comorbidities and predict mortality, with a higher score indicating a greater burden and greater risk of mortality [12]. A newer, validated bronchiectasis-specific comorbidity index, the Bronchiectasis Aetiology and Comorbidity Index (BACI) was also calculated [13].

Data on medicines regularly prescribed for patients by their general practitioner (GP), including drug name, prescribed dose and frequency of administration, quantity prescribed and date the drug was first and last issued, were collected. This information was used to compile a list of all medicines each patient was prescribed at the time of their clinic visit. Different strengths and formulations of the same medicine were counted separately.

The total number of prescribed medicines was determined by absolute count. Patients’ medication counts were used to identify whether or not three thresholds for polypharmacy (‘≥ 4 medicines’, ‘≥ 10 medicines’’ and ‘≥ 15 medicines’) had been exceeded.

MRCI scores were calculated using information collected about prescribed medicines [8]. The MRCI consists of 65 items divided into three sections: ‘Dosage forms’, ‘Dosage frequencies’ and ‘Additional instructions’. Each item is weighted according to the relative degree of complexity it contributes to the regimen [8]. Scores begin at 0 and because patients may be on any number of medicines, there is no maximum MRCI score. There are no widely recognised thresholds that represent particularly low, medium or high MRCI scores.

The primary outcomes of interest were oral antibiotic usage for suspected pulmonary exacerbations in the 6 months prior to clinic attendance, and IV antibiotic usage in secondary care in the 2 years prior to clinic attendance. Secondary outcomes included the total number of days’ treatment with IV antibiotics and admissions to hospital (all-cause and bronchiectasis-related) in the 2 years prior to clinic visit.

Medications prescribed for patients on an acute basis by GPs, including short courses of antibiotics, are recorded in patients’ ECRs. The following information regarding oral antibiotics prescribed for patients was recorded: issue date, antibiotic name and dose, frequency of administration, and duration prescribed (days). Indications for antibiotics are rarely specified on prescriptions issued in primary care. Oral antibiotic use for a pulmonary exacerbation was suspected if one or more of the following factors were present: the patient was prescribed an appropriate antibiotic (e.g., doxycycline, amoxicillin, co-amoxiclav, clarithromycin) for 14-days, and/or a sputum sample was collected and tested within a week of the prescription being issued [11]. Clinic notes were also consulted to check if the patient had reported any exacerbations since their last visit. In cases of ambiguity, the patients’ consultant physician provided clinical judgment of whether, or not, an antibiotic had been prescribed for a suspected pulmonary exacerbation. Only medicines prescribed in the past 6-months are available to view at the time of data collection, hence the 6-months time period for documenting oral antibiotic use.

IV antibiotic use was determined using data from patients’ discharge summary letters, which are available to view on the ECR and records extend back several years. Data regarding all IV antibiotics (date prescribed, name of antibiotic and duration of therapy) prescribed for patients for the treatment of an acute exacerbation of bronchiectasis and all hospitalisations in the past 2 years were collected. There was less ambiguity regarding the indication of IV antibiotics during hospital admissions because the indication of prescribed treatment during a hospital stay is routinely documented. IV antibiotic use and secondary outcomes were investigated over a longer period than oral antibiotic use to mitigate for seasonal variations in exacerbation rates.

A power calculation, based on a comparison of the mean number of times an oral antibiotic was prescribed for a suspected exacerbation in the preceding 6 months between patients who were prescribed 10 or more medicines and those who were prescribed less than 10 medicines, was performed prior to data collection. McCullough et al. [14] suggested a difference of two exacerbations within a 12-month period was importance clinically important difference. Assuming the standard deviation of exacerbations in 6 months was 1 (based upon findings from McCullough et al. [14]), with 100 patients, the power to detect a difference of one exacerbation within 6 months as significant at the 5% level was over 80%.

### Data analysis

All data were entered into IBM SPSS (Version 23). The Mann–Whitney U test was used to investigate if there was a significant difference in the primary and secondary outcomes of patients below and above the three thresholds for polypharmacy. Effect sizes (r) were calculated using the calculation ‘r = z/square root of N, where z is the standardized test statistic and N = number of cases’ [15].

Correlations between MRCI and the primary and secondary outcomes were investigated using Spearman rank-order correlation coefficient (Spearman’s correlation, *r*_*s*_). Correlations were considered strong/large if *r* = 0.5–1.0; moderate/medium if *r* = 0.3–0.5; weak/small if *r* = 0.1–0.3 [15].

To further investigate the association between polypharmacy and IV antibiotic use, patients were grouped into those who had had a course of IV antibiotics for the treatment of an exacerbation in the past 2 years and those who had not (status: did not receive IV antibiotics = 0, did receive antibiotics = 1). Chi square (*χ*^*2*^*)* tests were conducted to determine whether, or not, there was a difference in receipt of an IV antibiotic in the past 2 years between people who were above and below each of the three polypharmacy thresholds. Where significant differences were identified, unadjusted odds ratios were calculated. Other demographic and disease-related factors (age, gender, testing positive for *P. aeruginosa* or *H. influenzae*), and comorbidities may contribute to a higher exacerbation rate. Logistic regression was then used to adjust odds ratios for any other factors that could have potentially influenced the need for IV antibiotic use. The significance level was set at *p* ≤ 0.05 for all statistical tests. The associations between oral antibiotic use and polypharmacy were not investigated in this way because there was less certainty over the indication of oral antibiotic use.

## Results

Ninety-five patients were included in the study. Demographic and disease-related data of the sample population are outlined in Table 1.

**Table 1:** Demographic and disease-related data of sample population

Characteristic	Result
Gender	n (%)
Female	65 (68.4)
Male	30 (31.6)
Age	
Mean, y (SD)	62.6 (14.8)
FEV_1_ % predicted	
Mean (SD)*	80.9 (35.8)
Sputum microbiology	n (%)
Not recorded in past 6 months	30 (31.6)
*Pseudomonas aeruginosa*	17 (17.9)
*Haemophilus influenzae*	13 (13.7)
*Streptococcus pneumonia,*	1 (1.1)
*Staphylococcus aureus*	0 (0)
*Moraxella catarrhalis*	1 (1.1)
*Escherichia coli*	1 (1.1)
No significant growth	32 (33.7)
Number of comorbidities	n (%)
0	19 (20.0)
1	25 (26.3)
2	24 (25.3)
3	14 (14.7)
4	10 (10.5)
5	3 (3.2)
Median (IQR)	2 (2)
Charlson comorbidity index (CCI)	n (%)
0	52 (54.7)
1	25 (26.3)
2	11 (11.6)
3	3 (3.2)
4	3 (3.2)
5	1 (1.1)
Median (IQR)	0 (1)
Bronchiectasis aetiology and comorbidity index (BACI)	n (%)
Low risk (0)	55 (57.9)
Intermediate risk (1–5)	30 (31.6)
High risk (6 or more)	10 (10.5)
Median (IQR)	0 (5)

*SD* standard deviation, *IQR* interquartile range

*Mean FEV_1_ missing for 42 patients

Medication count per person ranged from 0 to 24 medicines. There was a median of 8 medicines per person and the interquartile range (IQR) was 7. Over three-quarters of patients in the sample were prescribed four or more medicines (n = 74; 77.9%), a third of patients were prescribed 10 or more medicines (n = 31; 33.0%), and 12 patients (12.8%) were prescribed 15 or more medicines. Total MRCI scores ranged from 0 to 68.5 and the median MRCI score was 26 (IQR 13.3–36.1).

Patients who were above the ‘≥ 4 medicines’ polypharmacy threshold had significantly more courses of oral antibiotics prescribed by their GP for a presumed pulmonary exacerbation in the past 6 months (effect size, r = 0.36; *p *< 0.001) and more admissions to hospital (all-cause) in the past 2 years (r = 0.32; *p *= 0.002) compared with patients who were below the threshold. There was no significant difference in IV antibiotic usage, duration of IV antibiotic therapy or admissions to hospital (bronchiectasis-related) across this threshold.

There were significant differences in all measured outcomes across the ‘≥ 10 medicines’ polypharmacy threshold. Effect sizes varied from weak to moderate, with the strongest effect size being for the number of admissions to hospital (all-cause) in the past 2 years (r = 0.4; *p *< 0.001).

At the ‘≥ 15 medicines’ threshold, the only significant difference was in the number of admissions to hospital (all-cause; r = 0.26; *p *= 0.011). Tables detailing computed U-statistics, effect sizes and p-values are provided in the electronic supplementary material.

There were significant positive correlations found between MRCI and all health outcomes. The strongest correlation was with the number of admissions to hospital for any reason in the past 2 years (*r*_*s*_= 0.413; *p *< 0.001), followed by the number of exacerbations managed with oral antibiotics in the past 6 months (*r*_*s*_= 0.318; *p *= 0.003). Correlation coefficients between MRCI and outcomes are outlined in the electronic supplementary material.

A significant difference was detected across the ‘≥ 10 medicines’ threshold (*χ*^*2*^= 4.912, *p *= 0.048) in the number of patients who had received an IV antibiotic in the past 2 years. The unadjusted odds ratio for the relationship was calculated by cross-tabulation to be 2.83 (95% CI 1.11–7.24).

Age, exceeding the ‘≥ 10 medicines’ polypharmacy threshold and *P. aeruginosa* status were included in the final logistic regression model (Table 2). Although included in the final model, age had no effect on whether or not patients had received IV antibiotics in the past 2 years (odds ratio 0.95, 95% CI 0.92–0.99). Exceeding the ‘≥ 10 medicines’ polypharmacy threshold significantly increased the likelihood that an IV antibiotic had been prescribed in the past 2 years when adjusted for age and *P. aeruginosa* status, compared with being prescribed less than 10 medicines (adjusted odds ratio 3.44, 95% CI 1.15–10.31).

**Table 2:** Adjusted odds ratios for factors found to significantly influence receipt of an intravenous antibiotic in the past 2 years, in a sample of patients with bronchiectasis

Factor	Odds ratio	95% confidence interval	Significance (*p*)
≥ 10 medicines	3.44	1.15–10.31	0.027
Age	0.95	0.92–0.99	0.010
*P. aeruginosa* positive	6.45	1.88–22.19	0.003

## Discussion

In this study of polypharmacy and MRC in patients with bronchiectasis, the prevalence of polypharmacy as defined by its lowest cited threshold was high. There was almost a four-fold difference in prevalence of polypharmacy in patients with bronchiectasis compared with the general population. The prevalence of polypharmacy, as defined using the ‘≥ 4 medicines’ threshold, amongst middle-aged people aged 45–65 years living in NI is 20.3% [2]. In an older cohort (over 70 years old) of patients living in UK, 23% of patients were prescribed four or more medicines [16]. The number and severity of comorbidities (as investigated using the CCI) were similar to the study presented in this paper [16].

MRCI scores ranged from 0 to 68.5, indicating that complexity varied substantially within the sample. The median MRCI score was 26, which was high in comparison to other MRC studies [10, 17, 18] and comparable to the findings of Negewo and colleagues who investigated MRCI in patients with COPD (median MRCI 24; IQR 18.5–31) [19]. MRCI is likely to be higher in people with respiratory disease due to the frequent use of different inhalation devices in these populations. Such formulations are heavily weighted in the MRCI [8]. Comparison of MRCI scores with other chronic respiratory diseases would help to inform the significance of the high MRCI determined in this study.

The high prevalence of polypharmacy and MRC prescribed for the sample population is concerning. These findings indicate that a considerable number of patients are at an increased risk of adverse drug events, potentially inappropriate prescribing and medication errors, which have been shown to be associated with polypharmacy [1, 2]. The findings of this study suggest that the prescription of 10 or more medicines is an appropriate threshold to prompt a medication review in patients with bronchiectasis. The role of pharmacists in medication reviews is well established and addition of regular pharmacy support to the bronchiectasis service may prove to be beneficial to patients [20].

This study also sought to investigate differences in oral and IV antibiotics use for across three different thresholds used to define polypharmacy. The ‘≥ 10 medicines’ threshold emerged as being the level at which, when exceeded, polypharmacy appeared more problematic for the study sample, based on the outcomes selected. There was a significant difference in all primary and secondary outcomes evaluated between patients below the ‘≥ 10 medicines’ threshold and those who exceeded it. That is, patients exceeding this threshold were prescribed oral and IV antibiotics more frequently, were admitted to hospital more often and received more IV antibiotics. Furthermore, patients who exceeded this threshold were found to be 3.44 times more likely to have required an IV antibiotic in the past 2 years when adjusted for *P. aeruginosa* infection.

### Strengths and limitations

This was a retrospective study and as such, precise measurement of pulmonary exacerbations was not possible. Instead, this study investigated antibiotic use for suspected pulmonary exacerbations and made assumptions with regards to the indication of oral antibiotics prescribed in primary care. These limitations, and those listed below, restrict the extent to which firm conclusions on the impact of treatment burden may be having on people with bronchiectasis. Although the findings of this study do not demonstrate causation, higher rates of antibiotic use, whether bronchiectasis-related or not, were observed in those patients who were prescribed polypharmacy and complex medication regimens. Conversely, antibiotics may have led to polypharmacy through treatment to combat adverse effects of those antibiotics. We did not consider appropriateness of polypharmacy in this study, and this is worthy of further exploration as patients with bronchiectasis are likely to be receiving multiple medications.

A sample size of 100 was calculated as having sufficient power (over 80%) in an independent samples *t* test that aimed to detect a difference of one exacerbation within 6 months as significant at the 5% level. The study achieved an adequate sample size of within 5% of the target (N = 95). Although consecutive sampling restricts the generalisability of the study, this method of sampling provided a useful illustration of the patient population attending the bronchiectasis clinic during the data collection period [21]. Furthermore, the demographics (age, gender, aetiology, sputum microbiology) of the sample population were found to be representative of the wider bronchiectasis population who are under specialist care [22, 23] The use of referenced thresholds for polypharmacy and validated tools (CCI, BACI and MRCI) enhances the validity of this study.

Due to the retrospective design of this study, many assumptions regarding medication use, both for regular medications and acute use of antibiotics were made. Non-adherence to medications, a known consequence of high treatment burden [3], was not considered. There were limitations to the information available on ECRs regarding the prescription of oral antibiotics by GPs. Only oral antibiotics that had been prescribed for patients in the past 6 months were available to view on the ECR. Furthermore, regular medicines prescribed by non-respiratory hospital specialists may have been missed during data collection. As such, the extent of polypharmacy and complexity of the medication regimens may have been underestimated. An accuracy check on data extraction was not performed due to restrictions in time and access to patient data. Findings may also have been affected by the time of year data were collected. Patients tend to exacerbate more frequently over winter than summer; therefore, a different pattern of oral antibiotic use may be observed if the study was repeated at a different time of year. Furthermore, we did not consider the impact of co-morbidities and the social environment on our findings.

## Conclusion

In conclusion, there is a high prevalence of polypharmacy and MRC in patients with bronchiectasis. Polypharmacy was associated with poorer outcomes when more than 10 medicines were prescribed, with patients exceeding this threshold being prescribed oral and IV antibiotics more frequently, being admitted to hospital more often and receiving more IV antibiotics. MRC was also associated with increased antibiotic use and more frequent admissions to hospital. The routine management of patients with bronchiectasis should consider the burden of treatment imposed on patients when reviewing outcomes.

## Electronic supplementary material

Below is the link to the electronic supplementary material.

Supplementary material 1 (DOCX 15 kb)
