# Supplementary material for: The association between polypharmacy and medication regimen complexity and antibiotic use in bronchiectasis
Source: Int J Clin Pharm. 2018 Jul 9;40(5):1342–8. doi: 10.1007/s11096-018-0681-1 (PMC6208574; doi:10.1007/s11096-018-0681-1)
Supplement: Supplementary file 1 — Supplementary material 1 (DOCX 15 kb) [file 11096_2018_681_MOESM1_ESM.docx]

International Journal of Clinical Pharmacy

The association between polypharmacy and medication regimen complexity and antibiotic use in bronchiectasis

Dr Maureen Spargo, PhD

School of Pharmacy, Queen’s University Belfast,

Prof. Cristín Ryan, PhD

The School of Pharmacy & Pharmaceutical Sciences

Panoz Institute, Trinity College Dublin

Dr Damian Downey, MD

Centre for Experimental Medicine, Queen’s University Belfast

Regional Respiratory Centre, Belfast City Hospital

**Corresponding author**

Prof. Carmel Hughes, PhD

[c.hughes@qub.ac.uk](mailto:c.hughes@qub.ac.uk)

| Outcome | ≥ 4 medicines threshold | | | ≥ 10 medicines threshold | | | ≥ 15 medicines threshold | | |
| --- | --- | --- | --- | --- | --- | --- | --- | --- | --- |
|  | Mann-Whitney  U | Effect size  r | p-value | Mann-Whitney  U | Effect size  r | p-value | Mann-Whitney  U | Effect size,  r | p-value |
| Oral antibiotic use in past six months | 401.5 | 0.36 | 0.000 | 709.5 | 0.24 | 0.019 | 439.5 | 0.07 | 0.494 |
| Intravenous antibiotic use in past two years | 664 | 0.14 | 0.184 | 707 | 0.30 | 0.003 | 373 | 0.19 | 0.066 |
| Duration of intravenous antibiotic therapy in past two years | 670 | 0.13 | 0.208 | 711.5 | 0.30 | 0.004 | 371.5 | 0.19 | 0.063 |
| Admissions to hospital in past two years (all-cause) | 454.5 | 0.32 | 0.002 | 542.5 | 0.40 | 0.000 | 290.5 | 0.26 | 0.011 |
| Admissions to hospital in past two years (bronchiectasis-related) | 601 | 0.20 | 0.057 | 735.5 | 0.25 | 0.014 | 359 | 0.19 | 0.061 |

**Table A: Comparison of primary and secondary outcomes above and below each polypharmacy threshold investigated**

| Outcome | Spearman’s correlation coefficient | Significance *(p)* |
| --- | --- | --- |
| Oral antibiotic use in past six months | 0.318 | 0.002* |
| Intravenous antibiotic use in past two years | 0.276 | 0.007* |
| Duration of intravenous antibiotic therapy in past two years | 0.271 | 0.008* |
| Admissions to hospital in past two years (all-cause) | 0.413 | 0.000* |
| Admissions to hospital in past two years (bronchiectasis-related) | 0.257 | 0.012 |
| *Correlation is significant at the *p*≤0.01 level. | | |

**Table B: Correlation between Medication Regimen Complexity Index and primary and secondary outcomes**
